# Polydopamine-Coated Natural Rubber Sponge for Highly Efficient Vapor Generation

**DOI:** 10.3390/polym14071486

**Published:** 2022-04-06

**Authors:** Han Yu, Yuqi Shi, Aiwu Ding, Jianhe Liao, Hongxing Gui, Yongping Chen

**Affiliations:** 1 School of Materials Science and Engineering, Hainan University, Haikou 570228, China; ch_yp@aliyun.com (H.Y.); shiyuqi888@aliyun.com (Y.S.); dingaw@hirub.com (A.D.); 990359@hainanu.edu.cn (J.L.); 2 Rubber Research Institute, Chinese Academy of Tropical Agricultural Sciences, Haikou 571101, China

**Keywords:** recycled NR sponge, polydopamine coatings, vapor generation

## Abstract

The global water crisis is becoming more and more serious, and solar steam generation has recently been investigated for clean water production and wastewater treatment. However, the efficiency of solar vapor transfer is still low. It is a great challenge to find photothermal materials which simultaneously have high energy transfer efficiency, facile production, and are low cost. To address this, we propose a method which is simple, low cost and suitable for large-scale preparation to fabricate the photothermal materials based on using recycled natural rubber sponge (NRS) coated with polydopamine (PDA). X-ray photoelectron spectroscopy analysis confirmed that when the PDA coated the surface of the NRS, the hydrophilicity of the sponge was significantly improved. Scanning electron microscopy characterization showed that the PDA-coated natural rubber sponge (PNRS) maintained the porous 3D skeleton of the pristine sponge. As a result, PNRS exhibits excellent photothermal properties, a very high evaporation rate of 1.35 kg m^−2^ h^−1^, and an energy transfer efficiency of 84.6% can be achieved under a light intensity of 1 sun (1 kW m^−2^). It is worth noting that the vapor generation of PNRS is still at a high level with 1.06 and 1.09 kg m^−2^ h^−1^ in the corrosive liquids of 1 M H_2_SO_4_ and 0.5 M NaOH, respectively. The photothermal materials based on using recycled NRS have good application prospects in seawater desalination and the purification of wastewater, which also provides a new method for the recycling of waste NRS.

## 1. Introduction

With increasingly serious environmental pollution, swift population growth, and the rapid development of the global economy, the shortage of water resources has become one of the biggest challenges facing mankind [1,2]. More and more people have difficulties surviving because of water shortages, and many countries have had the sustainable development of their economy seriously hindered because of this. If new solutions cannot be found, the problem of water shortage will continue to plague mankind [3,4,5]. Seawater is available in huge reserves, and even a small part of it can be used to fully alleviate the shortage of fresh water [6,7,8,9]. Seawater desalination methods mainly include distillation, reverse osmosis, and electrodialysis. Solar evaporation systems are very promising and sustainable desalination methods because of their green and renewable advantages, which are economical and effective ways to solve the problem of water shortages [10,11,12,13].

Compared with traditional membrane treatment or heat treatment, the solar evaporation system locally heats the material surface to generate steam and desalinate seawater instead of heating the bulk water [14,15,16,17]. To improve the efficiency of steam generation, it is necessary to fabricate the solar evaporation system reasonably. The first essential element is highly efficient solar absorbers [18,19,20,21]. Three kinds of solar absorbers, namely carbon-based [22,23,24], plasmonic-based [13,21,25], and semiconductor-based [26,27] absorbers, have been widely investigated in recent years. The hydrophilic porous structure of the materials ensures the transportation of water and the escape of steam, which can cause the photothermal materials to absorb solar energy and generate steam continuously [19,28], such as porous polymer [29,30,31]. Although these materials have achieved good results, they have several obvious problems, such as either being costly or non-renewable, and they are difficult to apply on a large scale [10,32].

Latex sponge is a kind of porous rubber material where the cell structure is all connected or mostly connected. NRS is prepared from natural rubber latex (NRL), a kind of biosynthetic polyisoprene. NRS products are becoming more and more popular because of many advantages, such as high elasticity, vibration absorption, light weight, buoyancy, cushioning performance, thermal and acoustic insulation, inertness, high porosity, and good aging properties. NRS has a 3D channel structure, which can effectively improve the evaporation rate of the solar evaporator [33,34,35,36]. In addition, the sponge has high mechanical stability and can be reused many times, which is conducive to reducing the cost. However, the sponge itself cannot effectively convert solar energy into heat, and water is difficult to convert into steam [33,37,38,39,40].

Polydopamine (PDA) has many excellent properties, such as good biocompatibility, good biodegradability, and excellent photothermal properties. PDA has strong adhesion and can combine with almost all substances through the self-polymerization of monomer dopamine with catechol groups [41,42,43,44]. At the same time, PDA is a kind of broadband solar absorption material, where the spectral absorption range includes ultraviolet, visible, and even near-infrared regions [45,46,47]. Therefore, PDA has been widely used as photothermal material for vapor generation due to its strong surface adhesion and efficient photothermal performance.

In this article, PDA-coated NRS was prepared via the self-polymerization of dopamine on the surface of the sponge. After PDA modification, not only does the 3D porous structure of PNRS remain unchanged, but also the hydrophilicity of the sponge is improved. This is beneficial for water to move to high-temperature regions through capillary action, and conducive to the rapid replenishment of evaporated water on the sponge surface [32,48]. To avoid heat loss, PNRS was placed on a PS foam connected to absorbent cotton threads [49,50], which prevented the PNRS from directly contacting a large amount of water so that it could quickly heat up, keep the water continuously transported to the evaporation interface, and generate more steam. This kind of solar evaporation device with high efficiency of photothermal conversion and excellent water absorption performance is not only simple to prepare, but also has high evaporation efficiency. It will be an effective and economical method to solve the shortage of water resources.

In China, hundreds of thousands of tons of NRS products are produced every year, which creates many sponge scraps. How to deal with these scraps is a huge challenge. It will be an effective way to kill two birds with one stone to recycle the sponge wastes into photothermal materials to solve the problem of water shortages.

## 2. Materials and Methods

### 2.1. Materials

The waste NRS products were supplied by Jiangsu Aidefu Latex Products Co., Ltd. (Taizhou, China). Dopamine hydrochloride was purchased from Macklin Biochemical Technology Co., Ltd. (Shanghai, China). Ammonia (17%) was provided by Guangzhou Jinhuada Chemical Reagent Co., Ltd. (Guangzhou, China). Ethanol, H_2_SO_4_, NaOH and NaCl were obtained from Xilong Scientific Co., Ltd. (Guangzhou, China). All chemicals were analytical reagents and used as received without further purification. The water used in the studies was ultrapure water with a resistivity of ≥18 MΩ cm provided by a Purifier Ultra-Pure Water System (Shanghai Fushite Co., LTD, Shanghai, China).

### 2.2. Preparation Process of PNRS

**Figure 1:**
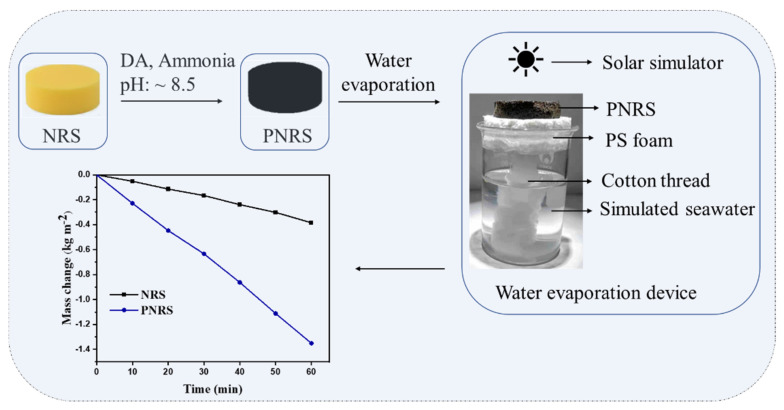
Process of solar evaporation of PNRS.

The preparation process of PNRS is shown in Figure 1. In a typical process, the NRS was cleaned in ethanol and distilled water using an ultrasonic system several times, 8 mg/mL aqueous solution of dopamine hydrochloride was added to the flask, and the pH was adjusted to 8.5 with ammonia. Then, the pretreated NRS was added to the solution and reacted at room temperature for 18 h. The PDA-coated NRS was obtained by washing with ethanol and drying to a constant weight in a vacuum-drying oven at 60 °C, and named PNRS.

### 2.3. Solar Evaporation Tests

Simulated seawater (3.5% NaCl) was prepared in a 100 mL beaker. The device for water evaporation is shown in Figure 1. The samples were irradiated by a solar simulator with an optical filter for the standard AM 1.5 G spectrum (CEL-HXF300, Beijing Zhongjiao Jinyuan Technology Co., Ltd., Beijing, China), and an optical power meter (CEL-NP2000, China) was used to calibrate the illumination intensity. The temperatures were measured by infrared thermal imager (HT-H8, Beijing Xinsite Co., Ltd., Beijing, China). The mass change in the liquids was monitored by electronic scales, and the evaporation rate and energy transfer efficiency were accordingly determined. The transfer efficiency (η) can be calculated via the following formula [5]:

(1) $$\eta =\frac{mh_{LV}}{I}$$

where *m* is the mass flux, *h_LV_* is the total enthalpy of liquid–vapor phase change, and *I* is the illumination intensity on the sample surface.

### 2.4. Characterization

The morphology of the sponges was observed with a field emission scanning electron microscope (FEI Nova Nano SEM 450, FEI, Hillsboro, OR, USA). X-ray photoelectron spectroscopy (XPS) analyses were carried out on an XPS spectrometer (Escalab250Xi, Thermo Fisher Scientific, Waltham, MA, USA). Contact angle measurements were performed using a SINDIN goniometer (SDC-100, Dongguan, China) with 5 µL of deionized water as the probe liquid. The optical absorption performance was measured by a UV-vis-NIR spectrometer (Shimadzu UV3600i plus, Kyoto, Japan) from 200 to 1500 nm.

## 3. Results and Discussion

### 3.1. SEM Analysis

**Figure 2:**
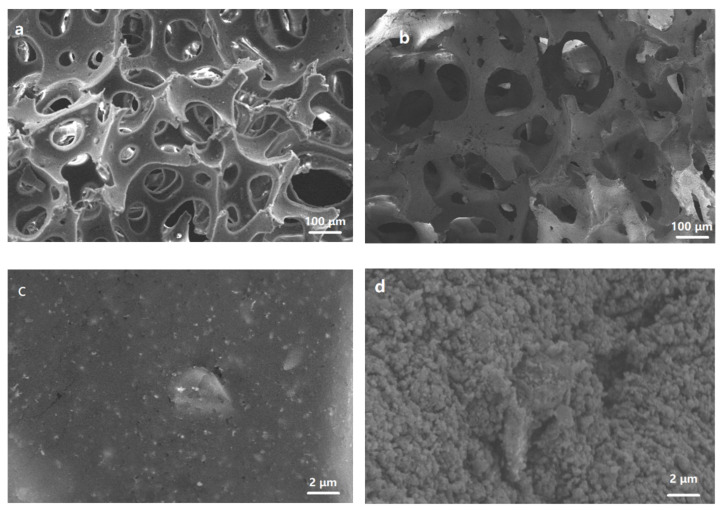
SEM photographs of NRS and PNRS ((**a**,**c**): NRS; (**b**,**d**): PNRS).

Scanning electron microscopy (SEM) images of NRS and PNRS are shown in Figure 2. NRS has a three-dimensional porous structure (Figure 2a), with pore sizes ranging from tens to hundreds of microns. PNRS has a similar porous structure (Figure 2b), which is very important for photothermal materials to transport water and escape steam. The enlarged image shows that the surface of the NRS skeleton is quite smooth (Figure 2c), whereas the surface of PNRS becomes rough after being coated with PDA (Figure 2d). The rough surface can reduce the reflection of sunlight and enable the solar absorber to harvest more energy.

### 3.2. XPS Analysis

**Figure 3:**
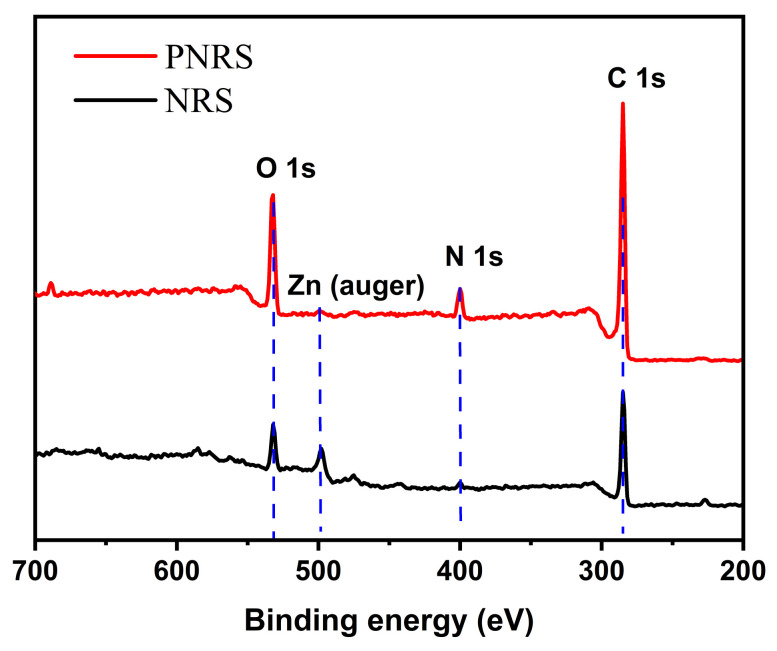
XPS curves of NRS and PNRS.

The elemental composition of NRS and PNRS surfaces was analyzed by XPS, as shown in Figure 3. For the NRS curve, the peaks at 284, 400, 498 and 545 eV belong to C1s, N1s, Zn (auger) and O1s, respectively. The N1s peak is very weak due to the small amount of protein existing in NRS, and the Zn (auger) peak is attributed to ZnO used as additive in the preparation of sponge. For the PNRS curve, the peak value of N1s increases significantly, which indicates that the content of element N on the surface of PNRS increases greatly owing to PDA being coated onto the sponge surface. In addition, the peak at 498 eV is almost disappeared, which can demonstrate that the PNRS surface is covered with a layer of PDA which contains no element Zn.

### 3.3. Water Contact Angle (CA) of NRS and PNRS

**Figure 4:**
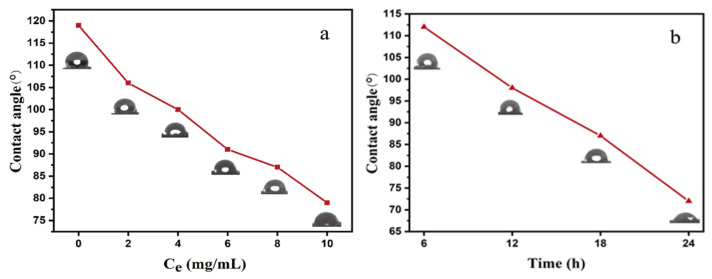
(**a**) CAs of NRS and PNRS coated with different concentrations of dopamine solution, (**b**) CAs of PNRS modified by different time (C_e_ = 8 mg/mL).

In general, hydrophilicity is important for photothermal materials to have a robust evaporation rate. NRS is hydrophobic with a contact angle of 119° because polyisoprene is a nonpolar polymer. After surface modification with different concentrations of the dopamine solution, the hydrophilicity of the PNRS surface was significantly improved. The CAs of the PNRS became smaller, indicating that the hydrophilicity of PNRS is better with a higher concentration of dopamine (Figure 4a) and a longer reaction time (Figure 4b). This demonstrates that CAs can be adjusted by changing the reaction conditions.

### 3.4. Optical Absorption Performance of NRS and PNRS

**Figure 5:**
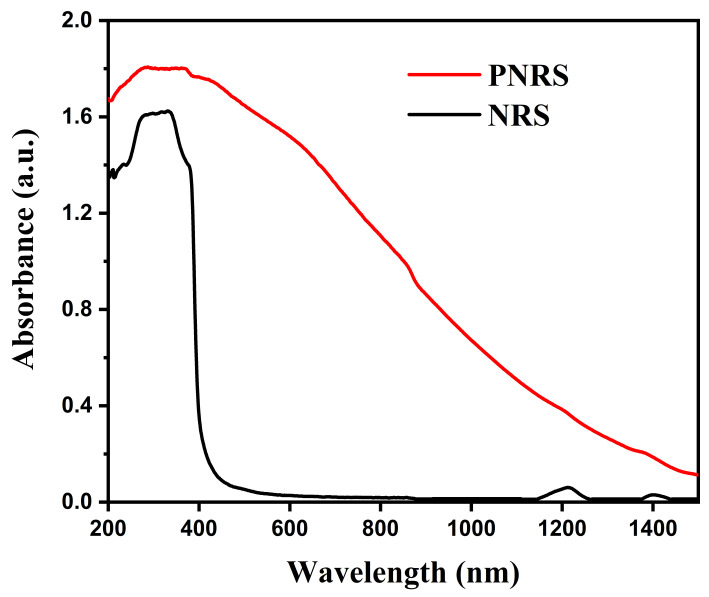
UV–Vis–NIR absorption spectra of PNRS and NRS.

A high optical absorption ability can improve the materials’ photothermal performance. The UV–Vis–NIR absorption spectra of NRS and PNRS were measured in the wavelength range of 200–1500 nm, as shown in Figure 5. The results show that PNRS has a wider absorption range and stronger absorbance than NRS from the ultraviolet to near-infrared region. NRS has almost no absorption in the visible and near-infrared region, and has only a certain absorption in the ultraviolet region. It can be predicted that PNRS has better photothermal performance after surface coating via PDA than NRS.

### 3.5. Evaluation of Photothermal Performance of NRS and PNRS

A suitable device is very important to enhance the evaporation efficiency. The device for solar evaporation in this study is shown in Figure 1. A piece of PNRS was placed on a PS foam with a hole in the middle, and a bunch of absorbent cotton thread was immersed into the water and connected to the PNRS through the hole [38]. Because of its hydrophilic and porous structure, PNRS can continuously transport water to the sponge surface via capillary action. The surface temperature of PNRS is much higher than that of the water body, so that the water evaporation is strengthened and a better evaporation effect is achieved [21,51].

**Figure 6:**
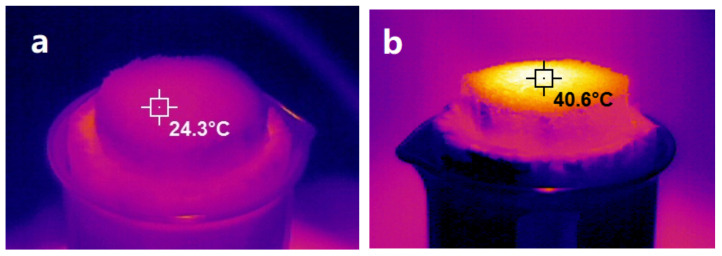

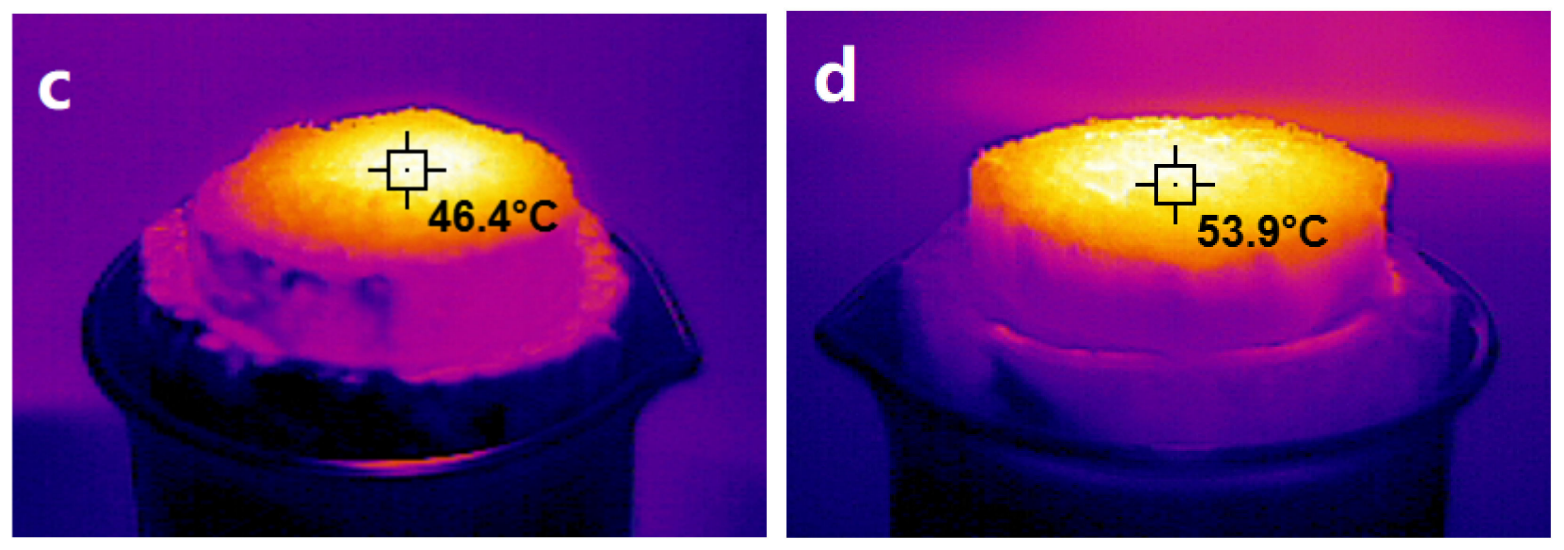
Infrared thermography of NRS ((**a**), 1 sun) and PNRS ((**b**), 1 sun; (**c**), 2 sun; (**d**), 3 sun).

Infrared thermography can directly measure the surface temperature of sponges, as shown in Figure 6. Under the illumination intensity of 1 sun, the surface temperature of NRS is only 24.3 °C, while that of PNRS is as high as 40.6 °C. With the increase in light intensity, the surface temperature of PNRS increases obviously, and reaches nearly 54 °C under 3 sun intensity. It is demonstrated that the PNRS surface can harvest more solar energy and reach higher temperatures after being coated with PDA.

**Figure 7:**
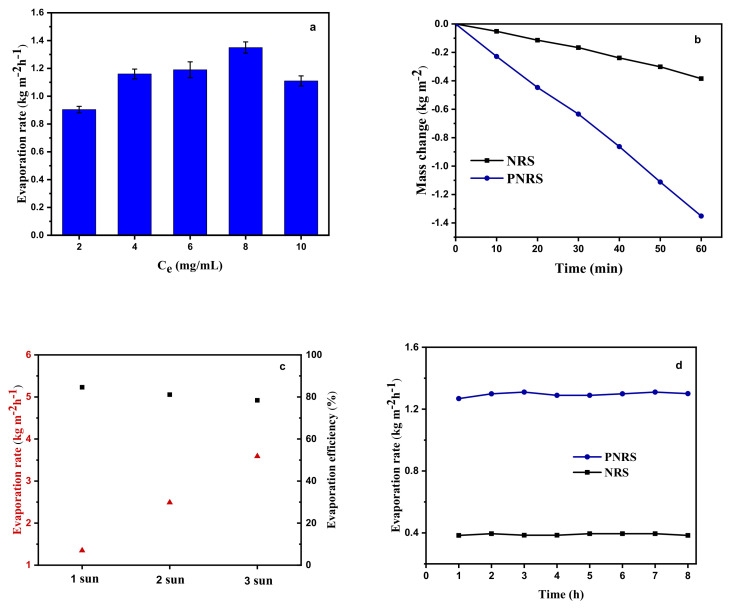
(**a**) Evaporation rate of PNRS modified with different concentrations of dopamine (1 sun); (**b**) water evaporation mass loss (1 sun); (**c**) evaporation rate and efficiency under different light intensities; (**d**) evaporation rate of PNRS with continuous desalination for 8 h (1 sun).

As shown in Figure 7a, the water evaporation rates of the sponges modified by different dopamine concentrations were investigated. With the increase in dopamine concentration, the evaporation rate accelerated, which could be due to the formation of a thicker PDA coating on the surface of the NR sponge. When the concentration of dopamine is 8 mg/mL, the evaporation rate with 1.35 kg m^−2^ h^−1^ is the highest under 1 sun intensity. The difference in water evaporation performance between NRS and PNRS (modified with dopamine concentration of 8 mg/mL) is shown in Figure 7b. The mass of water evaporation of NRS is only 0.38 kg m^−2^ within 1 h, while that of PNRS is as high as 1.35 kg m^−2^, which is more than 3.5 times that of NRS. In addition, under different light intensities (1 sun, 2 sun, 3 sun), the evaporation rate of PNRS is 1.35 kg m^−2^ h^−^^1^, 2.58 kg m^−2^ h^−1^ and 3.75 kg m^−2^ h^−1^, and the photothermal conversion efficiency is 84.6%, 81.1% and 78.4%, respectively (Figure 7c).

The long-term stability of the solar evaporator is necessary for desalination [52]. Salt accumulates on the evaporator surface, blocking both energy harvest and the channel for steam escape, resulting in a decline in the vapor generation performance [20]. The evaporation rate of PNRS is maintained at a high level with more than 1.3 kg m^−2^ h^−1^ for continuous steam generation within 8 h, indicating the good stability of the solar-driven desalination system (Figure 7d).

**Figure 8:**
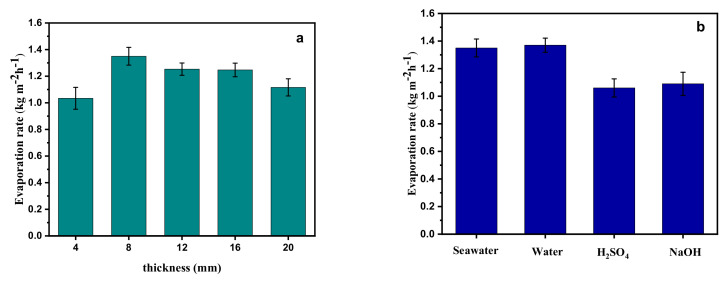
(**a**) Evaporation rate of PNRS with different thickness; (**b**) evaporation rate of PNRS in seawater, tap water, 1 M H_2_SO_4_ and 0.5 M NaOH.

Further study on the evaporation rate with different thicknesses of PNRS is shown in Figure 8a. The results show that the thickness has an obvious effect on the evaporation rate. With the thickness of 4, 8, 12, 16 and 20 mm, the evaporation rate of PNRS is 1.04, 1.35, 1.25, 1.24 and 1.12 kg m^−2^ h^−^^1^, respectively. When the thickness of the sponge is low, the absorbed solar energy increases with the increase in thickness. However, if the thickness exceeds 8 mm, the lower part of the sponge not only cannot absorb more solar energy, but also will block the heat transfer from top to bottom, resulting in a reduced evaporation rate.

In addition to desalination, the evaporation of corrosive liquids is also of great significance for pure water production and pollutant treatment [53]. We studied the water evaporation performance of PNRS in different liquids, including tap water, 1 M H_2_SO_4_, and 0.5 M NaOH solutions. As shown in Figure 7b, the evaporation rates in seawater, tap water, 1 M H_2_SO_4_, and 0.5 M NaOH solution are 1.35, 1.37, 1.06, and 1.09 kg m^−2^ h^−1^, respectively. The results show that strong acid and alkali solutions can slightly reduce the evaporation performance of PNRS, but the evaporation rate of the above two kinds of liquid remains at a high level, over 1.0 kg m^−2^ h^−1^, indicating that the photothermal materials based on PNRS can play an effective role in the treatment of corrosive solutions such as H_2_SO_4_ and NaOH.

## 4. Conclusions

PNRS, a PDA-coated sponge, was prepared through one-step polymerization of dopamine on the surface of recycled NRS. The preparation process of PNRS is facile, low-cost, and suitable for large-scale production. PNRS exhibits excellent photothermal properties because of its 3D porous skeleton and PDA coating. Moreover, PNRS not only possesses excellent desalination performance, but also has a high evaporation rate in corrosive liquids. It has good application prospects for desalination and wastewater treatment. It also provides a new idea and method for the recycling of waste NRS.

## Data Availability

All data are contained within the article.
